# Injections of Oil of Turpentine for the Radical Cure of Fistulæ

**Published:** 1886-03

**Authors:** 


### Medical News.

### Injections of Oil of Turpentine for the Radical Cure of Fistulæ.

Cecchini has employed oil of turpentine with good results in the treatment of several varieties of fistulæ. He claims that this substance acts as a powerful antiseptic, produces granulation, and can never do harm if a reasonable amount of care is exercised.

1. *Fistulæ in Ano.*—Seven cases treated, with five cures. The turpentine was injected by means of a syringe. As it causes considerable pain, it may be diluted with almond or olive oil. Short fistulæ are most easily cured by this remedy.

2. *Caries of Petrous Portion of Temporal Bone.*—Four cases cured in from two to three months. Boracic acid was used in conjunction with the turpentine. The discharge of fetid pus soon ceased, and complete cure rapidly followed.

3. *Dental Fistulæ.*—Eight fistulæ, with caries of alveolar process and maxillaries, were completely cured.

4. *Fistula of Steno's Duct.*—The fistula treated was caused by an abscess following a gun-shot wound. From the parotid region, fistulæ extended into the cheek, angle of jaw and neck. During mastication saliva flowed through the opening in the cheek. The fistula was healed in thirty days, with six injections.

Cecchini has also employed turpentine in the treatment of carbuncles and in post mortem wounds, in every case with excellent results.—*Centralblatt für Chirurgie, No. 1, 1886.*

---

Rubel reports a case of amaurosis produced by bromide of potassium. The patient was an epileptic woman, 23 years of age. From 2½ to 7 drachms of bromide were given daily, when amaurosis suddenly appeared. The bromide was stopped, and iodide of potassium given instead, when the visual disturbance disappeared. Bromide was again administered, causing a return of the amaurosis.—*Journal d'Oculistique.*

